# Remimazolam versus dexmedetomidine-based sedation for transcatheter aortic valve implantation: hemodynamic stability and early clinical outcomes

**DOI:** 10.3389/fcvm.2026.1925813

**Published:** 2026-09-21

**Authors:** Joon-Suk Bom, Ji-Won Yang, Ah Ran Oh, Jong-Hwan Lee, Jeong-Jin Min

**Affiliations:** 1 Department of Anesthesiology and Pain Medicine, Samsung Medical Center, Seoul, Republic of Korea; 2 Department of Anesthesiology and Pain Medicine, Samsung Medical Center, Sungkyunkwan University School of Medicine, Seoul, Republic of Korea

**Keywords:** aortic stenosis, conscious sedation, dexmedetomidine, hemodynamic instability, monitored anesthesia care, propensity score matching, remimazolam, transcatheter aortic valve implantation

## Abstract

**Background:**

Monitored anesthesia care (MAC) is increasingly used as part of minimalist transcatheter aortic valve implantation (TAVI) pathways, but evidence directly comparing remimazolam- and dexmedetomidine-based MAC with respect to intraprocedural hemodynamic stability and early clinical outcomes remains limited.

**Methods:**

In this retrospective, single-center, propensity score-matched cohort study, 433 patients who underwent TAVI under MAC between January 2021 and May 2026 were identified. After 1:1 propensity score matching, 264 patients were included, with 132 patients in each group. The primary outcome was intraoperative hemodynamic instability, defined as bradycardia requiring anticholinergic treatment or hypotension requiring vasoactive treatment. Other outcomes included rescue midazolam use, respiratory adverse events, and early postoperative clinical outcomes.

**Results:**

Remimazolam-based MAC was associated with less intraprocedural hemodynamic instability than dexmedetomidine-based MAC (53.8% vs. 69.7%; adjusted OR, 0.50; 95% CI, 0.31–0.82; *P* = 0.006), primarily reflecting less frequent vasoactive treatment for hypotension (50.8% vs. 67.4%; adjusted OR, 0.49; 95% CI, 0.30–0.81; *P* = 0.005). Bradycardia requiring anticholinergic treatment was numerically lower but not statistically significant. Rescue midazolam use was markedly lower with remimazolam (1.5% vs. 43.2%; *P* < 0.001). Respiratory adverse events, conversion to general anesthesia, postoperative delirium, length of stay, and 30-day clinical outcomes did not differ significantly between groups.

**Conclusions:**

In patients undergoing TAVI under MAC, remimazolam-based sedation was associated with a lower incidence of intraprocedural hemodynamic instability and less frequent rescue midazolam administration than dexmedetomidine sedation, without significant differences in early postoperative clinical outcomes.

## Introduction

Transcatheter aortic valve implantation (TAVI) has become an established treatment for severe aortic stenosis across an expanding spectrum of surgical-risk profiles (1, 2). In parallel with improvements in valve technology, procedural planning, and operator experience, anesthetic management for TAVI has shifted from general anesthesia toward a minimalist strategy using local anesthesia with conscious sedation or monitored anesthesia care (MAC) (3), which may facilitate early recovery and shorten procedural workflow (4, 5).

An ideal sedative regimen for TAVI under MAC should provide sufficient procedural comfort and immobility while preserving respiratory drive and maintaining cardiovascular stability (6, 7). Dexmedetomidine, a selective *α*2-adrenergic agonist, has been widely used for procedural sedation because it provides cooperative sedation with minimal respiratory depression (8, 9). However, dexmedetomidine can cause bradycardia and hypotension through sympatholytic and vagotonic effects, and inadequate sedative depth may require adjunctive sedatives or opioids during the procedure (9, 10).

More recently, remimazolam has been increasingly used as another sedative option for procedural sedation. Remimazolam is an ultra-short-acting benzodiazepine that is rapidly metabolized by tissue esterases and can be antagonized with flumazenil (11, 12). Owing to its predictable onset and offset, as well as its reported cardiovascular stability (13), remimazolam has emerged as a potential alternative sedative for high-risk or elderly patients undergoing procedural sedation (14, 15). In the setting of TAVI under MAC, remimazolam may offer practical advantages by providing less hemodynamic perturbation, reliable sedation, and rapid recovery (16).

Although several studies have compared anesthetic strategies for TAVI (17), evidence directly comparing remimazolam- and dexmedetomidine-based MAC remains limited. Previous comparative studies have mainly focused on recovery profiles, such as arousal time or timely intensive care unit discharge (16, 18), whereas data integrating intraprocedural hemodynamic stability, respiratory events, sedation efficacy and early postprocedural outcomes in real-world TAVI practice are still insufficient. Therefore, in this retrospective observational study, we aimed to compare remimazolam-based and dexmedetomidine-based MAC in patients undergoing TAVI, with particular focus on intraprocedural hemodynamic stability, sedation efficacy and peri-procedural clinical outcomes.

## Materials and methods

### Study design and patients

This study was conducted on patients who underwent TAVI at Samsung Medical Center, a tertiary care center in South Korea. All patients who underwent TAVI between January 2021 and May 2026 were evaluated for eligibility. Patients who underwent emergent TAVI (*n* = 6), those who received other sedative agents or underwent the procedure without sedation (*n* = 9) were excluded. The study data were obtained from the institutional clinical data warehouse. The study was approved by the Institutional Review Board of Samsung Medical Center (Approval number: SMC 2026-06-031-001), and the requirement for informed consent was waived owing to the retrospective nature of the study.

### Study period and institutional TAVI practice

TAVI was introduced at our institution in July 2010, and 180 procedures had been performed by the end of 2020. By September 2020, MAC had become the default anesthetic strategy for elective TAVI, and therefore the present study period began in January 2021, after substantial institutional TAVI experience had accumulated and the MAC-TAVI workflow had become established. The core interventional cardiology and cardiac anesthesia teams remained stable during the study period, with no major changes in the overall procedural workflow, including the predominant transfemoral access and temporary pacing strategy.

### Study exposure and perioperative management

Patients were classified according to the primary sedative strategy used for MAC: remimazolam-based MAC or dexmedetomidine-based MAC. Based on our institutional protocol, TAVI procedures were typically performed under MAC unless the patient's overall condition was unstable or a surgical approach was required. Before December 2022, dexmedetomidine was the primary sedative agent for TAVI under MAC; following an institutional practice change implemented in December 2022, remimazolam was adopted as the primary sedative agent. The institutional MAC-TAVI sedation strategies before and after this practice change are summarized in Supplementary Table S1.

Dexmedetomidine was initiated at 1 μg/kg/h and subsequently titrated to a maintenance infusion rate of 0.3–0.7 μg/kg/h. In the dexmedetomidine group, remifentanil (0.01–0.1 μg/kg/min) was selectively co-administered via continuous infusion at the discretion of the attending anesthesiologist, with no prespecified criteria governing its initiation and with the specific indication not systematically recorded. Midazolam was administered as a rescue sedative in incremental bolus doses when the intended level of sedation was not achieved. Remimazolam was administered as an initial bolus of 2–3 mg, followed by a continuous infusion typically maintained at 0.5 mg/kg/h, ranging from 0.2 to 0.7 mg/kg/h, in conjunction with remifentanil via continuous infusion (0.01–0.1 μg/kg/min) in all patients. Local infiltration with 1% lidocaine was performed by the operator after adequate sedation was confirmed by a Ramsay Sedation Scale score of ≥2, and sedation depth was continuously monitored and titrated at the discretion of the attending anesthesiologist to maintain clinically adequate sedation while preserving spontaneous respiration. Sedative and opioid infusions were discontinued after confirmation of prosthetic valve function. In patients receiving remimazolam, flumazenil was administered at the discretion of the attending anesthesiologist if residual sedation persisted at the end of the procedure.

Perioperative management adhered to institutional standards involving multidisciplinary risk stratification and perioperative planning in collaboration with the heart team. TAVI procedures were performed using a minimalist approach, without routine use of transesophageal echocardiography and with reliance on computed tomography-based pre-procedural planning. Through the transfemoral approach, a balloon-expandable valve (SAPIEN 3 or SAPIEN 3 Ultra; Edwards Lifesciences, Irvine, CA, USA) or a self-expandable valve (CoreValve Evolut R, Medtronic, Minneapolis, MN, USA; Navitor, Abbott, Santa Clara, CA, USA; or ACURATE neo2, Boston Scientific, Marlborough, MA, USA) was implanted according to aortic root anatomy and operator discretion. No cerebral embolic protection device was used. Transthoracic echocardiography was performed by the cardiology team immediately after the procedure to assess the implanted prosthetic valve function. The anesthesiologist performed radial arterial cannulation for continuous intra-arterial blood pressure monitoring and secured an additional peripheral intravenous catheter. An intraoperative mean arterial pressure (MAP) of ≥65 mmHg was generally targeted; phenylephrine or ephedrine was typically used for transient hypotension and norepinephrine for more persistent hypotension, with the choice and timing of vasoactive treatment determined by the attending anesthesiologist according to the clinical context. Supplemental oxygen was administered via a high-flow nasal cannula at 10 L/min, with the fraction of inspired oxygen (FiO₂) adjusted between 0.4 and 0.8 throughout the procedure.

### Study outcomes and definitions

The primary outcome was intraoperative hemodynamic instability, defined as the occurrence of either bradycardia requiring anticholinergic treatment or vasoactive treatment for hypotension during the procedure. Bradycardia was defined as a heart rate below 50 beats/min accompanied by administration of atropine or glycopyrrolate. Hypotension was defined as a mean arterial pressure (MAP) < 65 mmHg accompanied by vasoactive treatment with phenylephrine, ephedrine, or norepinephrine, representing clinically relevant hypotension requiring intervention. Hypotensive episodes occurring during rapid ventricular pacing (RVP) and the immediate post-pacing period were excluded from the definition of hemodynamic instability, because the expected decrease in blood pressure during and immediately after RVP typically recovers promptly after cessation of pacing and is generally not treated with vasoactive agents. Procedural success was defined as successful prosthetic valve deployment and completion of the index TAVI procedure.

Secondary outcomes included a composite hemodynamic outcome, postoperative delirium, rescue midazolam administration, conversion to general anesthesia, anesthesia duration, time to anesthesia offset, intensive care unit length of stay, and hospital length of stay. The composite hemodynamic outcome was defined as the occurrence of intraoperative bradycardia, intraoperative hypotension, temporary pacemaker requirement, postoperative hypotension, or postoperative vasoactive drug use. Postoperative hypotension or hemodynamic support was defined as a cardiovascular component of the Sequential Organ Failure Assessment (SOFA) score of ≥1, indicating a mean arterial pressure of <70 mmHg or the need for vasoactive or inotropic support. Postoperative delirium was defined as a positive Confusion Assessment Method for the Intensive Care Unit (CAM-ICU) assessment or documentation of delirium in the medical record. Anesthesia duration was defined as the interval from anesthesia start to anesthesia end. Time to anesthesia offset was defined as the interval from procedural completion to anesthesia end, calculated using timestamps documented in the electronic anesthesia record. Anesthesia end was defined as the documented time of patient transfer out of the procedure room.

Additional exploratory outcomes included intraoperative hypertension, respiratory adverse events, use of reversal or vasoactive drugs, intraoperative adverse events, postoperative hemodynamic support, extracorporeal membrane oxygenation, and temporary pacemaker-related outcomes. Intraoperative hypertension was defined as a systolic arterial pressure >180 mmHg accompanied by administration of an antihypertensive agent, specifically nicardipine or esmolol. Respiratory adverse events included desaturation, defined as a documented episode of SpO₂ < 90%, and airway intervention, defined as placement of an airway device documented in the intraoperative anesthesia record, including an oropharyngeal or nasopharyngeal airway, a supraglottic airway, or a tracheal tube. Because this category was defined by device placement, patients who subsequently required conversion to general anesthesia are also counted here. Tertiary outcomes included 30-day mortality, permanent pacemaker implantation within 30 days, acute kidney injury, 30-day cardiovascular readmission, stroke, and myocardial infarction. Acute kidney injury was defined according to the Kidney Disease: Improving Global Outcomes (KDIGO) creatinine criteria. Stroke was identified from event logs and intensive care unit computed tomography or magnetic resonance imaging reports, and myocardial infarction was identified from event logs and coronary angiography reports.

### Statistical analysis

As this was a retrospective study using all available data during the study period, no formal sample size calculation was performed. Of the 433 patients in the analytic cohort, 427 (98.6%) had complete data for all propensity-score covariates; six patients with one or more missing covariate values were excluded from the primary propensity-score analysis. Propensity scores were estimated in the complete-case cohort of 427 patients using multivariable logistic regression including age, sex, body mass index, hypertension, diabetes mellitus, chronic kidney disease, atrial fibrillation, coronary artery disease, left ventricular ejection fraction, hemoglobin, N-terminal pro-B-type natriuretic peptide, albumin, and ASA physical status classification, treated as a continuous variable. Patients receiving remimazolam-based MAC were matched 1:1 to those receiving dexmedetomidine-based MAC using nearest-neighbor matching without replacement. The matching procedure targeted the average treatment effect on the treated (ATT), with a caliper width of 0.1 standard deviations of the logit-transformed propensity score. Covariate balance was assessed using standardized mean differences, with absolute values below 0.1 considered indicative of adequate balance.

In the matched cohort, non-sparse binary outcomes were analyzed using covariate-adjusted logistic regression with cluster-robust standard errors accounting for matched subclasses. The rule for selecting adjustment covariates was prespecified as inclusion of covariates with residual imbalance after matching, defined as an absolute standardized mean difference ≥0.1; in the primary matched cohort, these covariates were hemoglobin, hypertension, chronic kidney disease, and coronary artery disease. Sparse binary outcomes, defined as total events fewer than 10 or a minimum treatment-by-outcome cell count fewer than 5, were analyzed using exact McNemar tests based on discordant pairs. Continuous outcomes were analyzed using covariate-adjusted linear regression with cluster-robust standard errors and are reported as adjusted mean differences with 95% confidence intervals; group medians with interquartile ranges are presented descriptively. To assess the robustness of the primary outcome, two sensitivity analyses were performed: propensity score matching after median imputation of missing baseline covariates and multivariable logistic regression in the unmatched complete-case cohort. First, to assess robustness to an alternative handling of the limited missing propensity-score covariate data, each missing propensity-score covariate value was imputed using the pooled-sample median for that variable; propensity scores were then re-estimated and matching was repeated using the full analytic cohort (*n* = 433), yielding a separate sensitivity-analysis matched cohort. Second, multivariable logistic regression was performed in the unmatched complete-case cohort (*n* = 427), including treatment strategy and all 13 propensity-score covariates. In the matched sensitivity analysis, the outcome model was adjusted for covariates with residual imbalance after that match, using the same absolute SMD ≥0.1 criterion. *P* values for individual components of the primary outcome and for secondary, tertiary, and additional exploratory outcomes were not adjusted for multiple comparisons and should be interpreted as exploratory. All analyses were performed using R version 4.6.0 (R Foundation for Statistical Computing).

## Results

### Population and characteristics

The medical records of 448 consecutive patients who underwent TAVI between January 2021 and May 2026 were reviewed retrospectively. Patients who underwent emergent TAVI (*n* = 6) or received other sedative agents or no sedation (*n* = 9) were excluded, leaving 433 patients in the analytic cohort (Figure 1). The baseline characteristics of the unmatched and matched cohorts are summarized in Table 1. The mean age was 82.2 ± 5.0 years, and 197 patients (45.5%) were women. In the analytic cohort, 296 patients (68.4%) received remimazolam-based MAC. Six patients had one or more missing propensity-score covariates (dexmedetomidine/remimazolam, *n* = 2/4), comprising seven missing values: body mass index (1/0), left ventricular ejection fraction (0/3), hemoglobin (0/1), and NT-proBNP (1/1); the primary complete-case propensity-score analysis therefore included 427 patients (dexmedetomidine, *n* = 135; remimazolam, *n* = 292). After 1:1 propensity score matching, 264 patients were included in the matched cohort, with 132 patients in each group. Propensity score matching improved baseline balance between the two groups, with standardized mean differences below 0.1 for most covariates; residual imbalance (absolute SMD ≥0.1) remained for hemoglobin, hypertension, chronic kidney disease, and coronary artery disease, which were included as covariates in the adjusted outcome models. Procedural success was achieved in 431 of 433 patients (99.5%). The two unsuccessful procedures occurred in the remimazolam group and were attributable to vascular access failure and procedural failure requiring conversion to surgical aortic valve replacement, respectively. No procedure was abandoned because of inadequate sedation, and neither unsuccessful procedure was documented as directly attributable to the sedative regimen.

**Figure 1:**
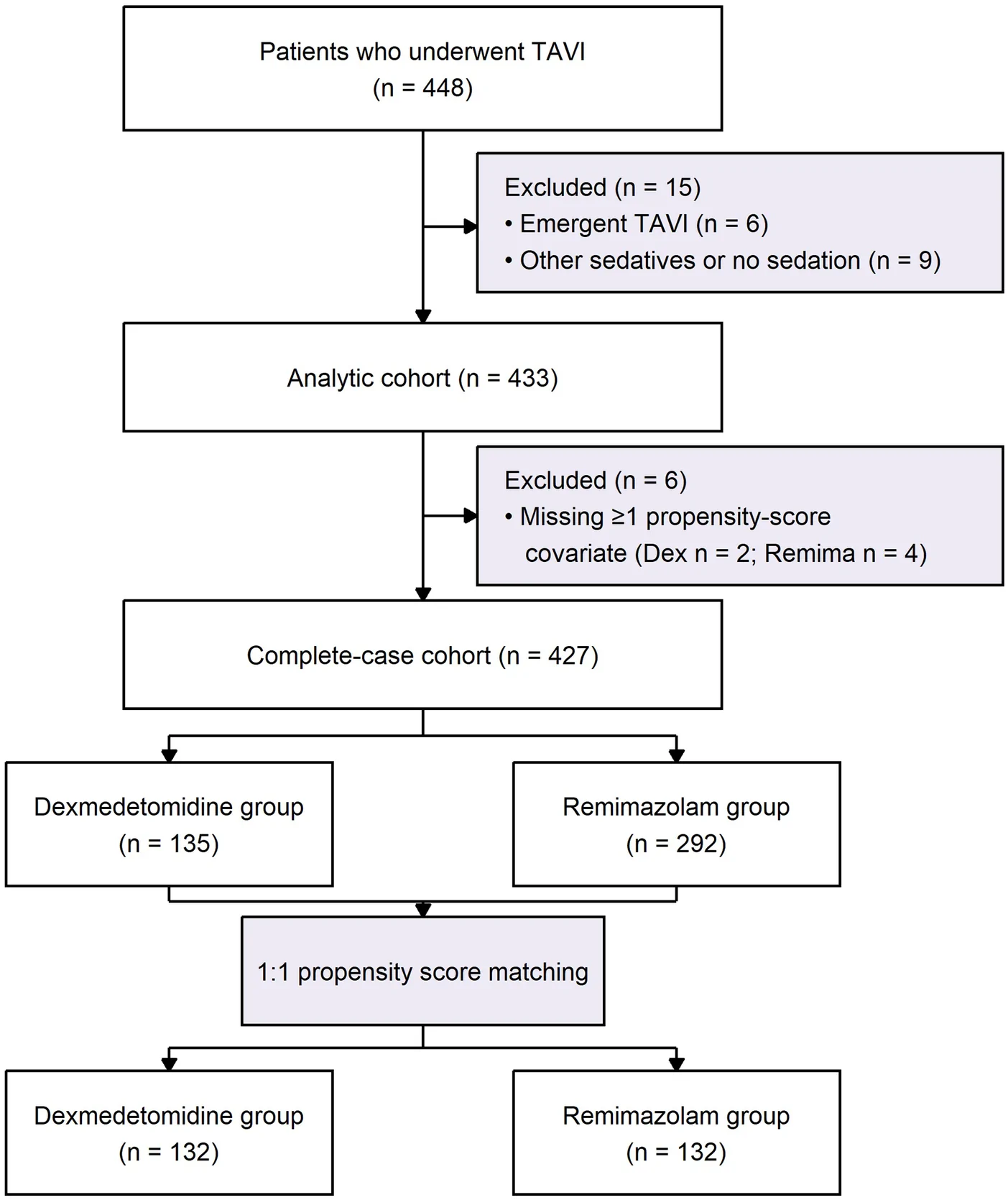
Study flow diagram. Dex, dexmedetomidine; Remima, remimazolam; PS, propensity score; TAVI, transcatheter aortic valve implantation.

**Table 1:** Baseline characteristics.

Variable	Before matching			After matching		
	Dex (*n* = 137)	Remima (*n* = 296)	Absolute SMD	Dex (*n* = 132)	Remima (*n* = 132)	Absolute SMD
Age, years	81.5 ± 4.6	82.5 ± 5.2	0.215	81.56 ± 4.66	82.05 ± 4.47	0.097
Female sex, *n* (%)	64 (46.7)	133 (44.9)	0.036	63 (47.7)	61 (46.2)	0.031
BMI, kg/m^2^	23.9 ± 4.1	23.9 ± 3.7	0.013	23.97 ± 4.10	24.04 ± 3.65	0.020
Hypertension	109 (79.6)	208 (70.3)	0.184	104 (78.8)	110 (83.3)	0.100
Diabetes mellitus	53 (38.7)	120 (40.5)	0.010	51 (38.6)	54 (40.9)	0.046
CKD	30 (21.9)	48 (16.2)	0.147	27 (20.5)	22 (16.7)	0.103
Atrial fibrillation	17 (12.4)	41 (13.9)	0.063	15 (11.4)	18 (13.6)	0.065
CAD	39 (28.5)	104 (35.1)	0.142	38 (28.8)	31 (23.5)	0.111
LVEF, %	59.8 ± 9.9	58.7 ± 12.0	0.111	60.11 ± 9.69	59.40 ± 12.25	0.060
Hemoglobin, g/dL	11.2 ± 1.7	11.6 ± 1.7	0.218	11.26 ± 1.64	11.46 ± 1.56	0.113
NT-proBNP, pg/mL	974 [342, 3796]	1185 [484, 3080]	0.082	859 [336, 3677]	999 [453, 2981]	0.003
Albumin, g/dL	3.9 ± 0.4	4.0 ± 0.3	0.082	3.97 ± 0.43	3.96 ± 0.34	0.014
ASA physical status	4.0 ± 0.1	4.0 ± 0.1	0.007	4.01 ± 0.09	4.01 ± 0.09	<0.001

Values are mean ± SD, median [IQR], or *n* (%), unless otherwise specified. Before-matching descriptive summaries are presented for the full analytic cohort (*n* = 433; dexmedetomidine, *n* = 137; remimazolam, *n* = 296), whereas before-matching absolute standardized mean differences (SMD) were calculated in the propensity-score complete-case cohort used for matching (*n* = 427; dexmedetomidine, *n* = 135; remimazolam, *n* = 292); six patients with one or more missing propensity-score covariates were excluded from the primary propensity-score analysis. After-matching descriptive summaries and SMDs are based on the matched cohort (*n* = 264; 132 per group). SMDs are absolute values from the MatchIt balance object. The adjustment set for outcome models (hemoglobin, hypertension, chronic kidney disease, coronary artery disease) was determined using unrounded post-match absolute SMD values ≥0.1. Dex, dexmedetomidine; Remima, remimazolam; BMI, body mass index; CKD, chronic kidney disease; CAD, coronary artery disease; LVEF, left ventricular ejection fraction; NT-proBNP, N-terminal pro-B-type natriuretic peptide; ASA, American Society of Anesthesiologists physical status.

### Intraoperative characteristics

Procedural and intraoperative characteristics are summarized in Table 2. Valve type distribution differed descriptively between groups, reflecting changes in device availability and institutional practice over the study period. Respiratory adverse events were infrequent. Intraoperative temporary pacemaker use was observed in 35 patients (26.5%) in the dexmedetomidine group and 23 patients (17.4%) in the remimazolam group. Intraoperative ECMO, transfusion, and CPR were infrequent. Remifentanil was co-administered in 82 of 132 patients (62.1%) in the dexmedetomidine group and in all 132 patients (100%) in the remimazolam group (Supplementary Table S1). Flumazenil was administered in 97 patients (73.5%) in the remimazolam group.

**Table 2:** Procedural and intraoperative characteristics in the matched cohort.

Variable	Dex (*n* = 132)	Remima (*n* = 132)	*P* value
Respiratory adverse events			
Desaturation (SpO₂ < 90%)	1 (0.8)	0 (0.0)	1.000
Airway intervention	8 (6.1)	9 (6.8)	0.822
Flumazenil use	0 (0.0)	97 (73.5)	<0.001
Intraoperative TPM	35 (26.5)	23 (17.4)	0.102
Intraoperative ECMO	4 (3.0)	0 (0.0)	0.125
Transfusion	3 (2.3)	0 (0.0)	0.250
CPR	2 (1.5)	2 (1.5)	1.000
Valve type, n (%)			
SAPIEN 3/SAPIEN 3 Ultra	87 (65.9)	83 (62.9)	—
CoreValve Evolut R	44 (33.3)	34 (25.8)	—
Navitor	0 (0.0)	12 (9.1)	—
ACURATE neo2	0 (0.0)	3 (2.3)	—
Indeterminate	1 (0.8)	0 (0.0)	—

Values are *n* (%). *P* values were obtained from covariate-adjusted logistic regression with cluster-robust standard errors accounting for matched subclasses for non-sparse binary outcomes and exact McNemar tests for sparse binary outcomes. Adjusted models included hemoglobin, hypertension, chronic kidney disease, and coronary artery disease. Valve type is presented descriptively and was not included in propensity score estimation or adjusted outcome models. *P* values were not adjusted for multiple comparisons and should be interpreted as exploratory. Dex, dexmedetomidine; Remima, remimazolam; SpO₂, peripheral oxygen saturation; TPM, temporary pacemaker; ECMO, extracorporeal membrane oxygenation; CPR, cardiopulmonary resuscitation.

### Primary and secondary outcomes

The primary and secondary outcomes in the matched cohort are shown in Table 3. In the unmatched analytic cohort, intraoperative hemodynamic instability occurred in 69.3% (95 of 137) of patients in the dexmedetomidine group and 53.4% (158 of 296) of patients in the remimazolam group. In the propensity score-matched cohort, the incidence of intraoperative hemodynamic instability was lower with remimazolam-based MAC than with dexmedetomidine-based MAC (71/132 [53.8%] vs. 92/132 [69.7%]; adjusted odds ratio, 0.50; 95% CI, 0.31–0.82; *P* = 0.006). This difference was mainly attributable to a lower incidence of hypotension requiring vasoactive treatment (50.8% vs. 67.4%; adjusted OR, 0.49; 95% CI, 0.30–0.81; *P* = 0.005), whereas bradycardia requiring anticholinergic treatment did not differ significantly between groups (3.8% vs. 8.3%; adjusted OR, 0.43; 95% CI, 0.14–1.25; *P* = 0.121). The composite hemodynamic outcome was also observed less frequently in the remimazolam group (62.1% vs. 74.2%; adjusted OR, 0.56; 95% CI, 0.34–0.94; *P* = 0.027).

**Table 3:** Primary and secondary outcomes in the matched cohort.

Outcome	Dex (*n* = 132)	Remima (*n* = 132)	Effect estimate [95% CI]	*P* value
PRIMARY				
Hemodynamic instability	92/132 (69.7%)	71/132 (53.8%)	0.50 [0.31–0.82]	0.006^a^
COMPONENTS OF THE PRIMARY OUTCOME				
Bradycardia	11/132 (8.3%)	5/132 (3.8%)	0.43 [0.14–1.25]	0.121^a^
Vasoactive treatment for hypotension	89/132 (67.4%)	67/132 (50.8%)	0.49 [0.30–0.81]	0.005^a^
SECONDARY (binary)				
Composite hemodynamic	98/132 (74.2%)	82/132 (62.1%)	0.56 [0.34–0.94]	0.027^a^
Rescue midazolam	57/132 (43.2%)	2/132 (1.5%)	—	<0.001^b^
GA conversion	3/132 (2.3%)	4/132 (3.0%)	—	1.000^b^
Delirium	20/132 (15.2%)	15/132 (11.4%)	0.75 [0.37–1.53]	0.434^a^
SECONDARY (continuous)				
Anesthesia duration, min	99 [91, 111]	92 [82, 105]	−7.95 [−12.40, −3.51]	0.001^c^
Time to anesthesia offset, min	8 [6, 12]	7 [6, 10]	−1.30 [−2.42, −0.19]	0.022^c^
ICU LOS, h	6 [6, 20]	6 [6, 19]	−0.83 [−6.89, 5.24]	0.788^c^
Hospital LOS, days	5 [3, 8]	4 [3, 7]	−0.87 [−2.28, 0.54]	0.227^c^

Effect estimates are calculated for the remimazolam group with the dexmedetomidine group as the reference. ^a^Adjusted logistic regression with cluster-robust standard errors accounting for matched subclasses; effect estimate is the adjusted odds ratio with 95% confidence interval. ^b^Exact McNemar test based on discordant matched pairs for sparse binary outcomes; *P* value only, effect estimate not estimable. ^c^Adjusted linear regression with cluster-robust standard errors accounting for matched subclasses; effect estimate is the adjusted mean difference with 95% confidence interval; medians [IQR] are shown descriptively. Adjusted models (a and c) included hemoglobin, hypertension, chronic kidney disease, and coronary artery disease. *P* values for individual components of the primary outcome and secondary outcomes were not adjusted for multiple comparisons and should be interpreted as exploratory. Dex, dexmedetomidine; Remima, remimazolam; CI, confidence interval; GA, general anesthesia; ICU, intensive care unit; LOS, length of stay.

Regarding secondary outcomes, rescue midazolam was administered significantly more often in the dexmedetomidine group than in the remimazolam group (43.2% vs. 1.5%; *P* < 0.001). Anesthesia duration was significantly longer in the dexmedetomidine group (99 [91–111] vs. 92 [82–105] min; adjusted mean difference, −7.95 min; 95% CI, −12.40 to −3.51; *P* = 0.001). Time to anesthesia offset was significantly longer in the dexmedetomidine group than in the remimazolam group (8 [6–12] vs. 7 [6–10] min; adjusted mean difference, −1.30 min; 95% CI, −2.42 to −0.19; *P* = 0.022). Postoperative delirium did not differ significantly between groups (15.2% vs. 11.4%; adjusted OR, 0.75; 95% CI, 0.37–1.53; *P* = 0.434). ICU length of stay and hospital length of stay did not differ significantly between groups.

### Tertiary and additional exploratory outcomes and sensitivity analyses

Regarding tertiary and additional exploratory outcomes, no significant between-group differences were observed in postoperative hypotension or hemodynamic support, ICU ECMO, temporary or permanent pacemaker-related outcomes, acute kidney injury, 30-day cardiovascular readmission, or 30-day mortality (Table 4). Among additional exploratory outcomes, intraoperative hypertension was observed more frequently in the dexmedetomidine group than in the remimazolam group (18.2% vs. 9.1%; adjusted OR, 0.44; 95% CI, 0.20–0.97; *P* = 0.041); as an exploratory outcome not adjusted for multiple comparisons, this finding should be interpreted with caution. Stroke was not observed in either group, and myocardial infarction occurred in 1 patient (0.8%) in the dexmedetomidine group and in none of the patients in the remimazolam group. The primary association was directionally consistent in both sensitivity analyses. After median imputation, propensity-score re-estimation, and rematching, the adjusted OR was 0.63 (95% CI, 0.39–0.99; *P* = 0.047; Supplementary Table S2). In the unmatched complete-case multivariable analysis, the adjusted OR was 0.51 (95% CI, 0.33–0.80; *P* = 0.003; Supplementary Table S2).

**Table 4:** Tertiary and additional exploratory outcomes in the matched cohort.

Outcome	Dex (*n* = 132)	Remima (*n* = 132)	Effect estimate [95% CI]	*P* value
TERTIARY				
30-day mortality	1/132 (0.8%)	1/132 (0.8%)	—	1.000^b^
Stroke	0/132 (0.0%)	0/132 (0.0%)	NE	—
Myocardial infarction	1/132 (0.8%)	0/132 (0.0%)	—	1.000^b^
PPM within 30 days	7/132 (5.3%)	13/132 (9.8%)	2.02 [0.79–5.16]	0.141^a^
CV readmission (30d)	3/132 (2.3%)	3/132 (2.3%)	—	1.000^b^
AKI (KDIGO)	9/132 (6.8%)	13/132 (9.8%)	1.95 [0.73–5.20]	0.183^a^
ADDITIONAL EXPLORATORY				
Intraoperative hypertension	24/132 (18.2%)	12/132 (9.1%)	0.44 [0.20–0.97]	0.041ᵃ
OR TPM retained	10/132 (7.6%)	13/132 (9.8%)	1.31 [0.55–3.14]	0.538^a^
ICU new TPM	2/132 (1.5%)	4/132 (3.0%)	—	0.625^b^
Postoperative hypotension or hemodynamic support	7/132 (5.3%)	10/132 (7.6%)	1.77 [0.66–4.77]	0.260^a^
ICU ECMO	3/132 (2.3%)	0/132 (0.0%)	—	0.250^b^

Effect estimates are calculated for the remimazolam group with the dexmedetomidine group as the reference. ^a^Adjusted logistic regression with cluster-robust standard errors accounting for matched subclasses; effect estimate is the adjusted odds ratio with 95% confidence interval. Adjusted models included hemoglobin, hypertension, chronic kidney disease, and coronary artery disease. ^b^Exact McNemar test based on discordant matched pairs for sparse binary outcomes; *P* value only, effect estimate not estimable. No formal statistical test was performed for stroke because no events occurred in either group. *P* values were not adjusted for multiple comparisons and should be interpreted as exploratory. Dex, dexmedetomidine; Remima, remimazolam; CI, confidence interval; NE, not estimable; OR, operating room; TPM, temporary pacemaker; ICU, intensive care unit; ECMO, extracorporeal membrane oxygenation; PPM, permanent pacemaker; CV, cardiovascular; AKI, acute kidney injury; KDIGO, Kidney Disease: Improving Global Outcomes.

## Discussion

In this retrospective study of patients undergoing TAVI under MAC, both remimazolam-based and dexmedetomidine-based sedation strategies were feasible, with high procedural success and no significant differences in early clinical outcomes. However, remimazolam-based MAC was associated with a more favorable intraprocedural management profile, as reflected by a lower incidence of hemodynamic instability and markedly less frequent rescue midazolam administration. The difference in the primary outcome was mainly attributable to a lower incidence of hypotension requiring vasoactive treatment, whereas bradycardia requiring anticholinergic treatment was numerically less frequent but not statistically significant. These findings suggest that, although both sedative strategies are clinically feasible for MAC-TAVI, remimazolam-based MAC may reduce the need for active intraprocedural rescue interventions.

The reduction in intraoperative hemodynamic instability should be interpreted primarily as a difference in intraprocedural management rather than as evidence of improved clinical outcomes. Patients undergoing TAVI are typically elderly and have severe aortic stenosis with limited cardiovascular reserve; therefore, anesthesiologists often need to respond promptly to decreases in arterial pressure or heart rate during the procedure. In this study, the difference in hemodynamic instability was mainly driven by fewer episodes of hypotension requiring vasoactive treatment, suggesting that remimazolam-based MAC was associated with less frequent active blood pressure rescue during the procedure. Although this may be relevant in a population with limited tolerance to hemodynamic perturbation, it did not translate into significant differences in postoperative cardiovascular support, length of stay, or early clinical outcomes. Similarly, bradycardia requiring anticholinergic treatment was numerically less frequent in the remimazolam group but did not differ significantly between groups. Therefore, remimazolam appeared to provide a smoother intraprocedural management profile, rather than a proven improvement in patient-centered outcomes.

In addition to this hemodynamic profile, remimazolam-based MAC was associated with markedly less frequent rescue midazolam administration. Because sedation depth was not directly quantified, this finding should be interpreted as an operational marker of sedation management rather than as validated evidence of superior sedative efficacy. The lower need for rescue sedation may partly reflect the procedural characteristics of TAVI and the combined remimazolam–remifentanil strategy used in this study. Percutaneous TAVI generally involves limited continuous surgical stimulation, but intermittent increases in noxious stimulation may occur during large-bore sheath manipulation and valve delivery. Dexmedetomidine may provide adequate cooperative sedation during less stimulating phases, but additional sedation may be required when procedural stimulation abruptly increases. By contrast, remimazolam was routinely combined with remifentanil in our institutional protocol because it lacks intrinsic analgesic properties, and this combined strategy may have provided more consistent hypnotic and analgesic coverage during periods of increased procedural stimulation. Thus, the lower need for rescue midazolam should be interpreted as a feature of the remimazolam-based MAC strategy rather than the isolated effect of remimazolam.

Our findings should be interpreted in the context of previous studies comparing remimazolam and dexmedetomidine for TAVI under MAC (16, 18). Prior studies have suggested that remimazolam may be associated with faster arousal and a favorable recovery profile compared with dexmedetomidine. A previous propensity score-matched study (16) reported lower postoperative requirements for vasoactive drugs and temporary pacemaker support in the remimazolam group. In the present study, remimazolam was similarly associated with favorable intraprocedural hemodynamic and sedation profiles; however, postoperative vasoactive drug use, temporary pacemaker-related outcomes, and other early clinical outcomes did not differ significantly between groups.

Several explanations may account for this discrepancy. First, the hemodynamic effects of both sedatives are generally short-lived and titratable, and any intraprocedural differences may have attenuated after discontinuation of sedation at the end of the procedure. Therefore, the advantage of remimazolam may be most apparent during active procedural management, whereas its effect on postoperative cardiovascular support may be limited. Second, differences between studies may reflect the retrospective nature of the data, including temporal changes in TAVI practice, patient selection, valve systems, pacing strategy, and institutional thresholds for vasoactive drug or temporary pacemaker use. Third, because TAVI-related conduction disturbance is strongly influenced by anatomical factors, valve type, implantation depth, and procedural characteristics, the choice of sedative alone may be insufficient to produce consistent differences in temporary or permanent pacing outcomes.

Our results suggest that remimazolam-based MAC should be viewed as a feasible alternative to dexmedetomidine-based MAC, rather than as a strategy proven to improve hard clinical outcomes. Its main advantage may lie in simplifying intraprocedural anesthetic management by reducing the need for hemodynamic rescue treatment and rescue sedation. This feature may be particularly relevant in elderly patients with limited cardiovascular reserve or in procedural settings where stable sedation and rapid adjustment of anesthetic depth are required.

## Study limitations

This study has several limitations. First, owing to its retrospective, single-center design, unmeasured confounding and selection bias cannot be excluded despite propensity score matching and covariate-adjusted analysis. Most importantly, the institutional sedative strategy changed over calendar time, with dexmedetomidine used predominantly before December 2022 and remimazolam thereafter. Although the study was restricted to a period after substantial institutional TAVI experience had accumulated, the MAC-TAVI workflow had been established, and the core procedural team was stable, institutional and operator experience inevitably continued to evolve over time. Moreover, because there was minimal temporal overlap between the two sedation strategies, the independent effects of sedative strategy and treatment era would not be reliably separable through additional adjustment for calendar time. Therefore, residual confounding from secular changes in procedural efficiency, perioperative practice, or hemodynamic management cannot be completely excluded. Second, the primary outcome incorporated treatment-based definitions, particularly the hypotension component, which required vasoactive treatment. Although an intraoperative MAP of ≥65 mmHg was generally targeted, the specific agent and timing of administration were left to the attending anesthesiologist's clinical judgment; therefore, no rigid drug-specific treatment protocol was used, and the outcome may have been influenced by individual anesthesiologist treatment decisions. In addition, archived blood pressure values were intermittent, so untreated brief episodes of MAP <65 mmHg could not be systematically and reliably ascertained retrospectively. Third, remifentanil was routinely co-administered in the remimazolam-based strategy but selectively in the dexmedetomidine-based strategy, and no prespecified criteria governed its initiation in the latter; the remimazolam and dexmedetomidine regimens therefore differed in their use of continuous remifentanil, so the comparison reflects two institutional sedation strategies rather than the isolated effect of either primary sedative, and the direction and magnitude of any influence from this asymmetric co-intervention cannot be determined. Fourth, time to anesthesia offset reflected the interval from procedural completion to the documented time of transfer out of the procedure room rather than the time of sedative discontinuation or complete recovery of consciousness, and may have been influenced by the asymmetric use of flumazenil, while the longer anesthesia duration in the dexmedetomidine group may also have reflected end-of-procedure workflow rather than sedation depth alone; the absolute between-group difference in time to anesthesia offset was small. Finally, sedation depth was not directly quantified; the secondary and tertiary outcomes were exploratory and not adjusted for multiple comparisons; and the remimazolam and dexmedetomidine regimens differed in their use of continuous remifentanil, so the comparison reflects two sedation strategies rather than the isolated effect of either sedative.

## Conclusion

Both sedation strategies were feasible for MAC-TAVI; however, remimazolam-based MAC was associated with fewer intraprocedural rescue interventions, although no significant differences were observed in early postoperative clinical outcomes.

## Data Availability

The datasets generated and/or analyzed during the current study are not publicly available due to institutional regulations and patient privacy restrictions. Data access is subject to approval by the Institutional Review Board and the institution. Requests to access the datasets should be directed to Jeong-Jin Min, mjj177@g.skku.edu.

## References

[1] Mack MJ Leon MB Thourani VH Makkar R Kodali SK Russo M. Transcatheter aortic-valve replacement with a balloon-expandable valve in low-risk patients. N Engl J Med. (2019) 380(18):1695–705. 10.1056/NEJMoa1814052 30883058

[2] Otto CM Nishimura RA Bonow RO Carabello BA Erwin JP Gentile F. 2020 ACC/AHA guideline for the management of patients with valvular heart disease: a report of the American College of Cardiology/American Heart Association joint committee on clinical practice guidelines. J Am Coll Cardiol. (2021) 77(4):e25–e197. 10.1016/j.jacc.2020.11.018 33342586

[3] Ajello V Fresilli S D'Amico F Franceschini G Aloisio T Gottin L. Management of anaesthesia for transfemoral transcatheter aortic valve implantation: an Italian interdisciplinary consensus statement. Br J Anaesth. (2026) 136(3):824–35. 10.1016/j.bja.2025.10.052 41483996

[4] Lauck SB Wood DA Baumbusch J Kwon J-Y Stub D Achtem L. Vancouver transcatheter aortic valve replacement clinical pathway: minimalist approach, standardized care, and discharge criteria to reduce length of stay. Circ Cardiovasc Qual Outcomes. (2016) 9(3):312–21. 10.1161/CIRCOUTCOMES.115.002541 27116975

[5] Lee SW Lee S Hahm KD. Postoperative pulmonary complications after transcatheter aortic valve implantation under monitored anesthesia care versus general anesthesia: retrospective analysis at a single large volume center. J Clin Med. (2021) 10(22):5365. 10.3390/jcm10225365 34830646 PMC8618621

[6] Sohn HM Ryu JH. Monitored anesthesia care in and outside the operating room. Korean J Anesthesiol. (2016) 69(4):319–26. 10.4097/kjae.2016.69.4.319 27482307 PMC4967625

[7] Song JW Soh S Shim JK. Monitored anesthesia care for cardiovascular interventions. Korean Circ J. (2020) 50(1):1–11. 10.4070/kcj.2019.0269 31642214 PMC6923237

[8] Candiotti KA Bergese SD Bokesch PM Feldman MA Wisemandle W Bekker AY. Monitored anesthesia care with dexmedetomidine: a prospective, randomized, double-blind, multicenter trial. Anesth Analg. (2010) 110(1):47–56. 10.1213/ane.0b013e3181ae0856 19713256

[9] Lee S. Dexmedetomidine: present and future directions. Korean J Anesthesiol. (2019) 72(4):323–30. 10.4097/kja.19259 31220910 PMC6676029

[10] Mayr NP Wiesner G Van Der Starre P Hapfelmeier A Goppel G Kasel AM. Dexmedetomidine versus propofol-opioid for sedation in transcatheter aortic valve implantation patients: a retrospective analysis of periprocedural gas exchange and hemodynamic support. Can J Anaesth. (2018) 65(6):647–57. 10.1007/s12630-018-1092-4 29464420

[11] Worthington MT Antonik LJ Goldwater DR Lees JP Wilhelm-Ogunbiyi K Borkett KM. A phase Ib, dose-finding study of multiple doses of remimazolam (CNS 7056) in volunteers undergoing colonoscopy. Anesth Analg. (2013) 117(5):1093–100. 10.1213/ANE.0b013e3182a705ae 24108261

[12] Kilpatrick GJ. Remimazolam: non-clinical and clinical profile of a new sedative/anesthetic agent. Front Pharmacol. (2021) 12:690875. 10.3389/fphar.2021.690875 34354587 PMC8329483

[13] Antonik LJ Goldwater DR Kilpatrick GJ Tilbrook GS Borkett KM. A placebo- and midazolam-controlled phase I single ascending-dose study evaluating the safety, pharmacokinetics, and pharmacodynamics of remimazolam (CNS 7056): part I. Safety, efficacy, and basic pharmacokinetics. Anesth Analg. (2012) 115(2):274–83. 10.1213/ANE.0b013e31823f0c28 22190555

[14] Lee M Lee C Choi GJ Kang H. Remimazolam for procedural sedation in older patients: a systematic review and meta-analysis with trial sequential analysis. J Pers Med. (2024) 14(3):276. 10.3390/jpm14030276 38541018 PMC10971009

[15] Rex DK Bhandari R Lorch DG Meyers M Schippers F Bernstein D. Safety and efficacy of remimazolam in high risk colonoscopy: a randomized trial. Dig Liver Dis. (2021) 53(1):94–101. 10.1016/j.dld.2020.10.039 33243567

[16] Kim J-H Nam J-S Seo W-W Joung K-W Chin J-H Kim W-J. Effects of remimazolam versus dexmedetomidine on recovery after transcatheter aortic valve replacement under monitored anesthesia care: a propensity score-matched, non-inferiority study. Korean J Anesthesiol. (2024) 77(5):537–45. 10.4097/kja.24138 39039823 PMC11467503

[17] Hung K-C Chen J-Y Hsing C-H Chu C-C Lin Y-T Pang Y-L. Conscious sedation/monitored anesthesia care versus general anesthesia in patients undergoing transcatheter aortic valve replacement: a meta-analysis. Front Cardiovasc Med. (2022) 9:1099959. 10.3389/fcvm.2022.1099959 36704470 PMC9872395

[18] Kitaura A Tsukimoto S Sakamoto H Hamasaki S Nakao S Nakajima Y. A retrospective comparative study of anesthesia with remimazolam and remifentanil versus dexmedetomidine and remifentanil for transcatheter aortic valve replacement. Sci Rep. (2023) 13(1):17074. 10.1038/s41598-023-43895-0 37816802 PMC10564871

